# Trends of recanalization therapies and state of art for ischemic stroke treatment in Campania region, Italy

**DOI:** 10.1007/s10072-022-06321-3

**Published:** 2022-09-08

**Authors:** Emanuele Spina, Paolo Candelaresi, Giampiero Volpe, Florindo D’Onofrio, Daniele Spitaleri, Gioacchino Martusciello, Giovanni Piccirillo, Francesco Briganti, Mario Muto, Michele Feleppa, Marco Sparaco, Andrea Manto, Teresa Cuomo, Salvatore Ascione, Patrizia Ripa, Daniele Giuseppe Romano, Vincenzo Andreone, Fiore Manganelli, Rosa Napoletano

**Affiliations:** 1 Department of Neurosciences, Reproductive and Odontostomatology, University of Naples “Federico II”, Via Pansini, 5, Naples, Italy; 2 Neurology Unit, P.O. “San Leonardo”, Castellammare Di Stabia, Italy; 3 Neurology and Stroke Unit. AORN “Antonio Cardarelli”, Naples, Italy; 4 Neurology and Stroke Unit, AOU “San Giovanni Di Dio Ruggi d’Aragona”, Salerno, Italy; 5 Neurology and Stroke Unit, AORN “San Giuseppe Moscati”, Avellino, Italy; 6 Neurology and Stroke Unit, AORN “Sant’Anna E San Sebastiano”, Caserta, Italy; 7 Department of Advanced Biomedical Sciences, University of Naples “Federico II”, Naples, Italy; 8 Diagnostic and Interventional Neuroradiology, AORN Antonio Cardarelli, Naples, Italy; 9 Neurology and Stroke Unit, AORN “San Pio”, Benevento, Italy; 10 Neuroradiology Unit, P.O. “Umberto I”, Nocera Inferiore, Italy; 11 Neurology and Stroke Unit, P.O. “Umberto I”, Nocera Inferiore, Italy; 12 Neurology and Stroke Unit, P.O. “Ospedale del Mare”, Naples, Italy; 13 Department of Diagnostic and Interventional Neuroradiology, University Hospital “San Giovanni Di Dio E Ruggi d’Aragona”, Salerno, Italy

**Keywords:** Stroke, Epidemiology, Campania, Trend, Recanalization therapies

## Abstract

**Background:**

According to the last Italian report by the Ministry of Health in 2018, the estimated number of acute ischemic strokes (AIS) in Campania is 10,000/year, with an expected number of 1390 intravenous thrombolysis (IVT) and 694 mechanical thrombectomies (MT). In 2017, only 1.5% of expected patients received IVT and 0.2% MT. This study analyzed the trend of IVT and MT in 2019–2020 and depicted the state of art of Stroke Care in Campania.

**Methods:**

From the regional health task force, we obtained the hospital discharge forms from all private and public hospitals in Campania; we selected patients with a principal diagnosis of AIS and measured the rate of patients admitted to neurology units and the rate of IVT, MT, and IVT + MT for both 2019 and 2020.

**Results:**

In 2019, we observed 4817 admissions for AIS; 2858/4817 (59.3%) patients were admitted to neurology units. Out of 4817 patients, 192 received IVT, 165 MT, and 131 IVT + MT (488 treated patients; 10.1%). In 2020, we observed 4129 admissions for AIS; 2502/4129 (62.7%) patients were admitted to neurology units. Out of 4129 patients, 198 received IVT, 250 MT, and 180 IVT + MT (628 treated patients; 15.2%). These results showed that despite a reduction of AIS admissions in 2020, the relative and absolute rate of recanalization treatments increased. However, the number of patients who were not admitted to neurology units nor received acute treatments remained dramatically high.

**Conclusion:**

Despite the development of acute treatments, the Campania Stroke Network still needs significative efforts to improve.

## Introduction

**Fig. 1:**
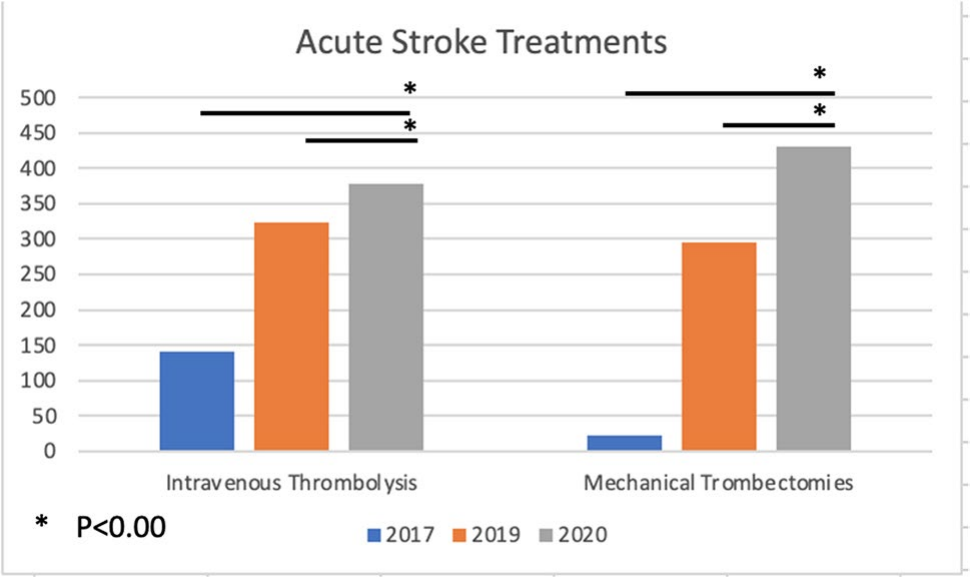
Reperfusion therapies in 2017, 2019, and 2020

Stroke is the second leading cause of death and the third cause of combined death-disability across the world [1]. In Italy, we estimated about 100,000 acute ischemic strokes per year (accounting for 62.4% of total strokes) [1, 2], with one million people suffering from residual disability. Back in 2003, the Ministry of Health licensed intravenous thrombolysis (IVT) in Italy, and from 2015, mechanical thrombectomy (MT) was approved as a recanalization strategy for large vessel occlusions (LVOs), coupled with (and not alternative to) IVT, unless contraindicated [3, 4]. Simultaneously, the Italian Stroke Organization and the Ministry of Health started an activity of continuous monitoring with periodical reports on patients’ outcome and organization of local systems of care (including rates of hospitalization, distribution of Stroke units, and number of acute treatments), referring the Italian stroke care to the aims established in the SAP-E (Stroke Action Plan for Europe) [5, 6]. With approximately 6 million inhabitants, Campania is the third most populous and the first densely populated region in Italy. The number of any stroke expected per year is approximately 14,000, 9500 of them being ischemic [7]. According to the last Italian report released by the Ministry of Health in 2018, every year in Campania, at least 1390 ischemic stroke patients should receive IVT and 694 MT. In 2017, only 157 (1.6%) AIS patients were treated with reperfusion therapies: 1.5% and 0.2% of expected patients received IVT and MT, respectively (Fig. 1) [8]. Moreover, only four hospitals were ready to administer IVT and only one could guarantee MT on a 24/7 basis. This study aimed to evaluate the hospitalization rate of ischemic strokes, the admissions to neurology units, and the trend of IVT and MT in 2019 and 2020.

## Methods

We obtained from the informatics service of Campania regional health task force the hospital discharge forms (properly anonymized) from all regional public and private hospitals, coded as “DRG 14,” corresponding to any ischemic or hemorrhagic strokes. We selected patients with a principal diagnosis of ischemic stroke, obtaining the total number of events. Then, we measured (1) the rate of patients admitted to neurology units in the whole region, in each local health service (“Azienda Sanitaria Locale”) and each independent hospital, excluding duplicates or post-acute admissions in rehabilitation units; and (2) the rate of IVT, MT, and IVT + MT for both 2019 and 2020 (Table 1). IVT was identified by the code 9910 (“Infusion of a thrombolytic agent”) as principal or secondary intervention. MT were identified either by the code 3810 (Endarterectomy), or 3811 (“Endarterectomy of intracranial vessel”), or 3812 (“Endarterectomy of head or neck vessel”), or 8841 (“Cerebral arteries arteriography”), or 0061 (“Percutaneous angioplasty/atherectomy of extracranial precerebral vessels”), or 3974 (“Endovascular removal of head and neck vessel obstructions”), or 0062 (“Percutaneous angioplasty/atherectomy of intracranial vessels”), or 0063 (“Stenting of the carotid artery”), or 0064 (“Percutaneous insertion of stent in extracranial precerebral vessel”), or 0065 (“Percutaneous insertion of intracranial vascular stent”), as the principal or secondary intervention. According to interventional codes, patients were classified as “IVT alone,” “MT alone,” or “bridging (IVT + MT) treatment.” Furthermore, we compared data from local registry datasets on recanalization therapies of 6 principal hospitals (AORN Cardarelli – Napoli; San Giovanni di Dio e Ruggi d’Aragona – Salerno; Umberto I – Nocera, Salerno; Sant’Anna e San Sebastiano – Caserta; San Giuseppe Moscati – Avellino; Ospedale del Mare—Napoli) with official data received from the regional health task force. In order to guarantee homogeneity, we used official data from the regional informatics service to evaluate the trend of recanalization therapies in 2019 and 2020. Statistical analysis was performed using STATA 13 software.

**Table 1:** Total stroke, total AIS admissions, and admissions in neurology units in 2019 and 2020

		Expected	2019	2020
Total stroke (AIS + ICH)		~ 10,000*	7709	6585
AIS admissions		~ 8000*	4817	4129
Hospitalization rate (on total population)		~ 7200*	0.7/1000	0.7/1000
Province	Naples		0.7/1000	0.6/1000
	Salerno		1.1/1000	0.9/1000
	Avellino		1.1/1000	1/1000
	Benevento		0.8/1000	0.7/1000
	Caserta		0.8/1000	0.7/1000
Admissions in neurology units (% on total admissions)		~ 7200*	2858 (59.3%)	2502 (57.9%)
Province	Naples		1159 (54%)	1028 (55.6%)
	Salerno		865 (71%)	718 (72.9%)
	Avellino		421 (92%)	360 (87%)
	Benevento		131 (58%)	97 (48%)
	Caserta		282 (37%)	299 (43%)

## Results

In 2019, we registered 7709 total admissions for acute stroke, 4817 (62.5%) of them being ischemic. The admission rate was 0.7/1000 inhabitants; 2858 (59.3%) AIS patients were admitted to a neurological unit. The highest rate of admissions was observed in the province of Avellino (1.1/1000 inhabitants), along with the highest rate of patients admitted to neurology units (92% of total AIS). The lowest rate of admissions was observed in the province of Naples (0.7/1000 inhabitants), whereas the lowest rate of patients admitted to neurology units was observed in the province of Caserta (37% of total AIS). According to the official data from the regional task force, in 2019, 488 patients (10.1% of total AIS) received an acute treatment: 192 patients received IVT alone, 165 MT alone, and 131 IVT + MT. Overall, 323/488 (66.1%) patients received IVT and 296/488 (60.6%) MT. The six principal hospitals accounted for 95% of total IVT (307/323) and 96% of total MT (286/296) treatments performed in Campania during 2019.

Compared to 2017, in 2019, we observed an increase of 130% in IVT treatments, 1186% in thrombectomies, and 210% in overall treatments. Sixty hospital wards reported at least one admission for AIS (32 in the province of Naples, 12 in the province of Salerno, 3 in Avellino, 4 in Benevento, 10 in Caserta). Neurology units regularly admitting AIS were 16 (8 in the province of Naples, 4 in Salerno, 2 in Avellino, 1 in Benevento, and 1 in Caserta). Stroke units with continuous highly intensive acute stroke care delivered by dedicated personnel were two (AORN Cardarelli and San Giovanni di Dio e Ruggi d’Aragona).

In 2020, we observed 6585 admissions for acute stroke, 4129 (62.7%) of them being ischemic. The admission rate was 0.7/1000 inhabitants; 2502 (57.9%) AIS patients were admitted to a neurological unit. The highest rate of admissions was again in the province of Avellino (1/1000 inhabitants), along with the highest rate of admissions to neurology (87% of AIS). The lowest rate of admissions was still in the province of Naples (0.6/1000 inhabitants), as well as the lowest rate of patients admitted to neurology was still recorded in the province of Caserta (43% of total AIS). According to the official data from the regional task force, in 2020, 628 patients (15.2% of the total AIS) received an acute treatment: 198 patients received IVT alone, 250 MT alone, and 180 IVT + MT. Overall, 378/628 patients (60.1%) received IVT and 430/628 (68.7%) MT. The six principal hospitals accounted for 96% of total IVT (363/378) and 98% of total MT (422/430) performed in Campania during 2020.

According to the datasets from the six principal hospitals, 585 patients received acute treatments in 2019 (+ 97, + 19.8% more than official data, accounting for 12.1% of total AIS) and 675 in 2020 (+ 47, + 7.4%, more than official data, accounting for 15.6% of total AIS). Compared to 2019, in 2020, we observed 14% fewer admissions for both any stroke (− 1124 patients admitted) and AIS (− 688), despite a similar rate of incidence of AIS (62% of the global number of stroke) and admissions to neurology units (about 60%). Regarding recanalization therapies, compared to 2019, in 2020, we observed a statistically significant increase in the global number of acutely treated patients (+ 28.6%, *p* < 0.00), as well as in the number of both IVT (+ 17.3%, *p* < 0.00) and MT (+ 45%, *p* < 0.00). The rate of admissions to neurological wards and the number of active stroke units did not differ (Table 2).

**Table 2:** Active stroke units and number of reperfusion treatments in 2017, 2019, and 2020

	Expected	2017	2019	Comparison 2019–2017	2020	Comparison 2020–2019	Statistical significance (2020 vs 2019)	Comparison 2020–2017 (var % rel)
1 level SU active	9	3	4	+ 1	4	equal		equal
2 level SU active	7	1	2	+ 1	2	equal		+ 1
Total treated patients (% of total)	NA	157	488 (10.1%)	+ 331 (+ 210%)	628	+ 140 (+ 28.6%)	(*p* < 0.00)	+ 431 (+ 274%)
Total IVT treatment (with bridging)	1390	140	323	+ 183 (+ 130%)	378	+ 55 (+ 17.3%)	(*p* < 0.00)	+ 238 (+ 170%)
Total MT treatment (with bridging)	694	23	296	+ 273 (+ 1186%)	430	+ 134 (+ 45%)	(*p* < 0.00)	+ 417 (+ 1813%)

## Discussion

Campania has shown a rapid development of recanalization treatments for AIS in barely few years. In fact, just in 2017, reperfusion treatment rates were comparable to those reported from Western European countries in the distant early 1990s [9]. Noteworthy, in just 3 years, IVT treatments triplicated (from 140 in 2017 to 378 in 2020), MT increased by 18 times (from 23 in 2017 to 430 in 2020), and the global number of treated patients was quadruplicated (from 157 in 2017 to 628 in 2020). Moreover, compared to the last report of stroke care in Italy for 2017, the number of hospitals delivering acute stroke treatments has increased from 4 to 6: half of them is active on a 24/7 basis for both IVT and MT, and the others are available only on daytime. Compared to the official regional data, the local registries of these six hospitals revealed an even higher number of recanalization treatments, in a range between 8 and 20% (up to 585 in 2019 and 675 in 2020), probably due to erroneous completion of hospital discharge forms. The emerging trend from this analysis is therefore consistent with a clear, progressive, and significant improvement of stroke treatments in Campania. We must mention, as an active contributor to these improvements, the Angels Initiative, launched in 2016 to create a global network of stroke-ready hospitals. In our territory, Angels worked as a link between territory, population, hospitals, and local health authorities contributing to raise population awareness, to improve training of healthcare professionals and to stimulate the sensibleness of health authorities to create a stroke network in Campania [10].

However, much remains to be done. The global number of stroke patients admitted to neurology units and the absolute number of acutely treated patients are respectively 30% and 10% lower than expected.

**Fig. 2:**
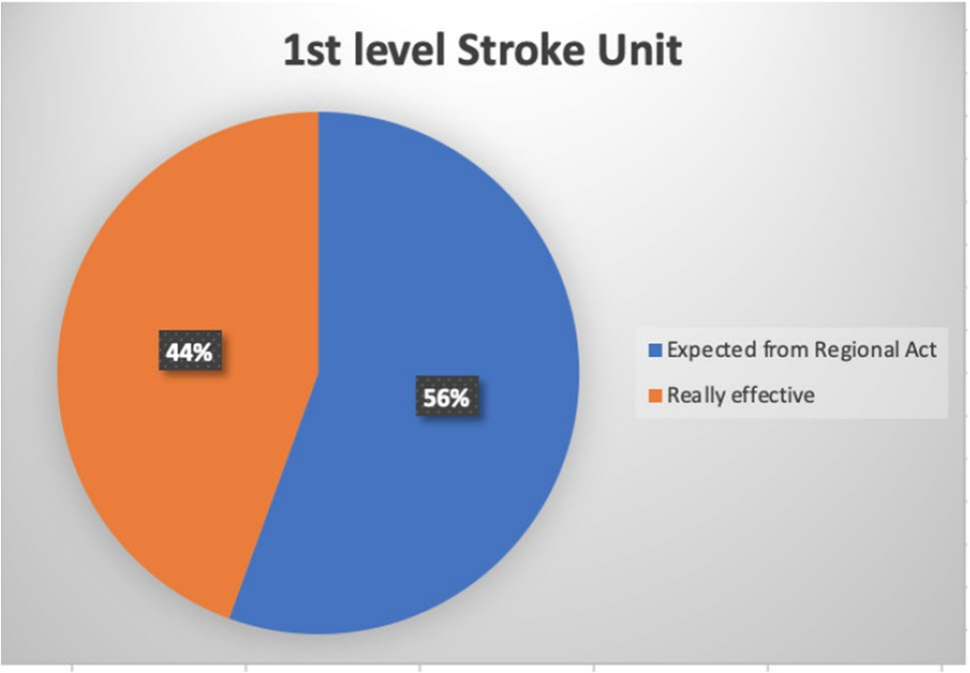
Percent of stroke unit of 1st level on the expected total from the Regional Act

**Fig. 3:**
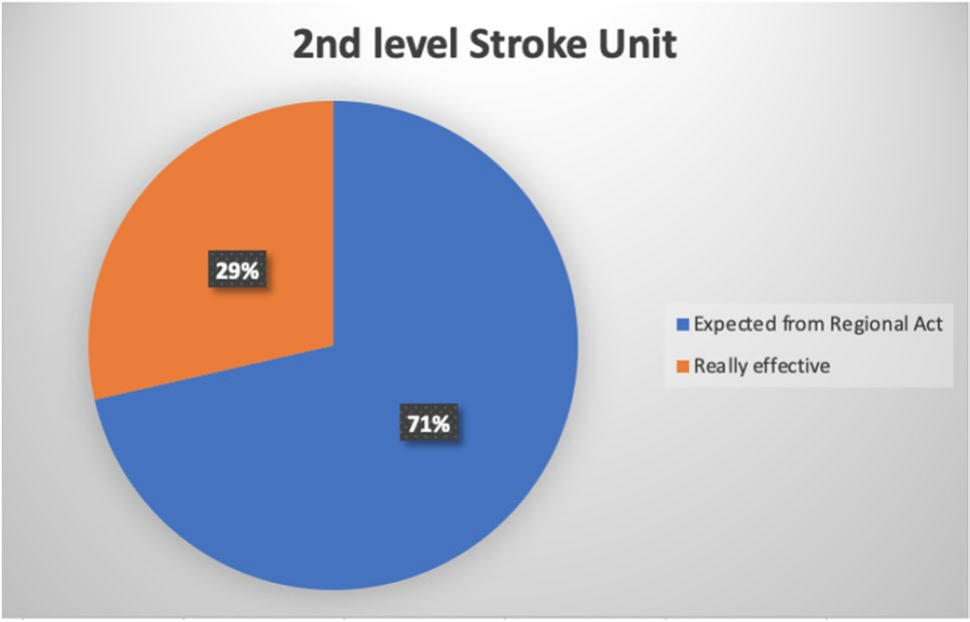
Percent of stroke unit of 2nd level on the expected total from the Regional Act

**Fig. 4:**
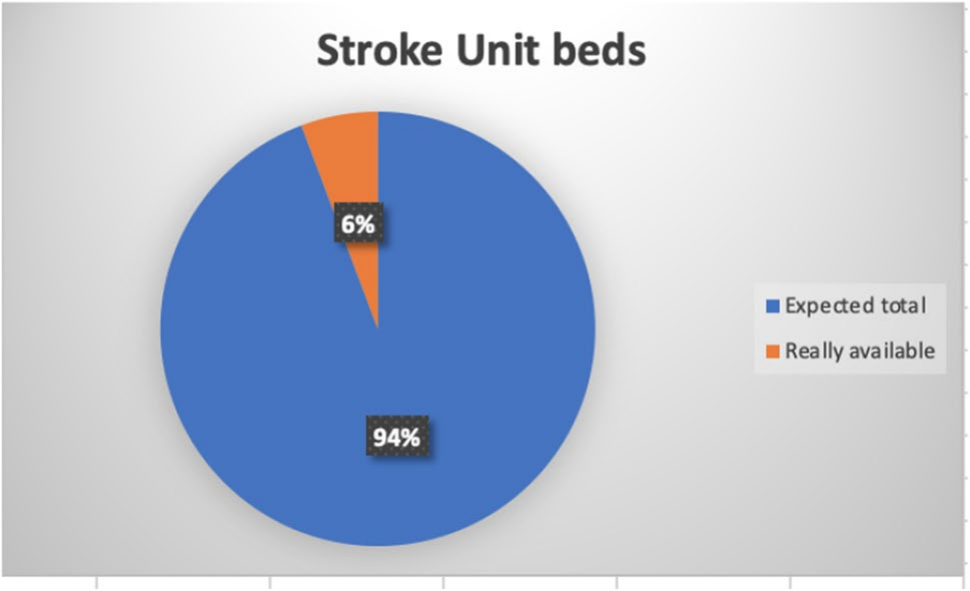
Percent of high-intensive monitored beds in neurology units on the expected total

The Act number 70/2015 by the Italian Government states that a primary stroke center should serve an area of 150,000–300,000 inhabitants, supported by a comprehensive stroke center for every 600,000–1,200,000 inhabitants [11], guaranteeing 1 stroke unit bed for every 19,000 inhabitants approximately. Campania Regional Government in the Regional Act number 63/2019 identified 9 primary stroke centers, 7 comprehensive stroke centers, and 11 hospitals for subacute stroke patients, located according to geographical distribution (maximum primary transport duration of 60 min) (Figs. 2 and 3) [12]. Unfortunately, at the moment, only 2 comprehensive stroke centers are actually active on a 24/7 basis, together with 4 neurology units serving as primary stroke centers, accounting for 18 stroke unit beds (Fig. 4). Therefore, the number of patients admitted to a stroke unit is dramatically low and far from the goal of 90%, as established in the Stroke Action Plan for Europe 2018–2030 [6].

The possible reasons are various: firstly, the patients’ unawareness of stroke symptoms determines a late call for help. According to a 2014 survey, among high-income countries, the Italian population is one of the less aware of stroke symptoms and its potential treatments [13]. Campaigns aimed at increasing lay people’s awareness of stroke care are needed, as this may possibly impact clinical outcome [13–16]. A possible useful model to improve stroke awareness in Campania population is what proposed in Emilia Romagna in 2017 in which, after a preliminary phase of assessment of main knowledge deficiencies in the general population, a group of physicians created a communicative strategy tailored on the needs of their community, using multilevel communication channels to promote preparedness, efficacy, and rapid actions [17].

Then, the lack of neurology units determines a lack of specific neurological care: with local political support, it is essential to reach the established regional requirements in order to guarantee modern, specialized, and qualified stroke care.

Moreover, an inadequate organization of regional stroke pathways is somehow confounding: particularly concerning for the emergency medical service is skipping closer hospitals, supposedly primary stroke centers, but actually still inactive. This may lead to either excessive delays in centralizing acute stroke care, contributing to the dispersion of admissions, accumulating delays, and limiting the chances of acute treatment, or to unnecessary congestion of comprehensive stroke centers. A real implementation of the regional stroke network, according to the Regional Act number 63/2019, is therefore urgently needed, creating 7 new primary stroke centers and 3 new comprehensive stroke centers, to be staffed by at least 9 and 12 stroke neurologists, respectively.

Lastly, specific programs aimed at training emergency healthcare personnel and qualifying young neurologists in stroke care are also urgently needed in order to increase prehospital recognition of stroke symptoms and improve the stroke chain of recovery.

Despite large vessel occlusions account for only 24 to 38% of ischemic stroke, in our region, MT increased significantly more than IVT and, surprisingly, in 2020, the relative rate of MT exceeded that of IVT [18]. This may reflect the lack of recognition of milder symptoms, the lack of primary stroke centers limiting the spreading of thrombolysis in peripheral areas, the difficulties, and delays in inter-hospital transfers (possibly worsened by pandemic) [19, 20], limiting the access to treatments mainly to more severe stroke with wider treatment windows. However, as recently stated in a meta-analysis of 5 RCT trials, the non-inferiority of direct MT compared to the combined IVT + MT approach has not been demonstrated, and therefore by far, IVT should not be skipped [21].

A potential turning point for the development of a stroke network in our region may be the activation of a task force aimed at continuous monitoring of quality indicators, as it still exists in other Italian regions, to further improve stroke care. The most commonly identified quality indicators are as follows: the rate of reperfusion treatments, the rate of admissions to stroke units, the use of international scales, as the NIHSS and mRS, the rate of patients undergoing adequate neuroimaging, the rate of patients receiving early rehabilitation and adequate secondary stroke prevention programs [22]. Developing shared stroke registries and joining international quality assessment programs may further improve efficacy in stroke care as shown in other countries [23–26].

In conclusion, stroke care in Campania is improving, thanks to the efforts and abnegation of healthcare professionals. However, a regional implementation of the stroke system of care is urgently needed, in order to guarantee treatments to the highest number of patients in the shortest time, and reduce the heavy personal, familial, social, and economic consequences of stroke, for the vast majority of patients and not only for lucky minorities.
